# Sea-Sickness

**Published:** 1897-09-18

**Authors:** 


### The Hospital

A Journal of

The Medical Sciences and Hospital Administration.

Vol. XXII.—No. 573.] [Sept. 18, 1897.

### SEA-SICKNESS.

Great multitudes of English folk, more or less weary and exhausted with many months of hard work, are still entering upon or returning from holidays which involve "going down to the sea in ships," and the majority of them must expect, unless they be exceptionally favoured by weather or by constitution, to suffer more or less from the malady which reduces the average Frenchman to a condition of indescribable limpness, and renders him an object of compassion, not only on the pier of Dover or Folkestone, but even when the spires of London are coming into view. It is, therefore, desirable for all whose journeys will take them beyond the boundaries of Britain to consider in what way their sufferings from sea-sickness, if they cannot be altogether prevented, may yet be reduced in duration and in severity; and it happens, more or less fortunately, that two great authorities have delivered themselves quite recently upon this question. Dr. Lauder Brunton, in his lectures on the action of medicines, devotes some pages, and Dr. Stocker, the medical officer to the Board of Trade at Glasgow, in the third volume of the "System of Medicine," edited by Professor Clifford Allbutt, devotes an entire article to the consideration of the subject. The views of these two physicians would be eminently helpful and satisfactory if it were not for the trifling consideration that they are almost diametrically opposed to one another, apparently in principle, and certainly in the practical applications of remedies to the relief of suffering. Each of them is entitled to a respectful hearing, Dr. Lauder Brunton as an experienced traveller by sea and land, Dr. Stocker as a former member of the medical staff of the Cunard Company.

Dr. Lauder Brunton places his main reliance upon bromide of potassium, with the proviso that it should be largely diluted. His plan is to put half a drachm into a bottle of soda water as soon as he goes on board, and to swallow the whole of it, repeating the dose, in a voyage of any length, apparently as often as may be required. Dr. Lauder Brunton also lays stress upon the usefulness of a firm abdominal bandage, which should be so arranged as to hold up the lower viscera against the diaphragm. This recommendation is one which we can entirely confirm, together with the statement that it is best to use for the purpose one of the long Oriental sashes called "cummerbunds," which are made of silk, and are at once light, long enough to take several turns round the abdomen, and strong enough to afford the necessary firmness of support. Dr. Stocker indicates a way in which the same end may be attained, we think somewhat less certainly, by other means. All slightly experienced sailors know that the most trying moment on shipboard is that of the descent of the vessel in her pitch or roll, at which time the sensations of the novice would leave him to believe that the foundation of the abdominal viscera had been suddenly abstracted, and that they were about to fall *en masse* into an abyss. This sensation is naturally attended by closure of the glottis and suspended inspiration, and Dr. Stocker says that the remedy for the discomfort which it entails is to be found in a volitional control of this closure, by means of a full inspiration taken deliberately and rhythmically with each descent. The necessary effort would also, we imagine, have the beneficial effect of occupying the attention, and thus of withdrawing it from sensations which are all the more apt to become overwhelming the more they are dwelt upon as harbingers of what is to follow them. Dr. Stocker disapproves of sedatives, and recommends chiefly, for those who are prone to suffer, careful regulation of the dietary and bowels for a day or two before a voyage; a good meal with some porter before going on board, and the recumbent posture with the head low until something like acclimatisation to the new conditions is attained. We are inclined to suggest a combination of the two methods—the dietary and the laxative as a preliminary for those who can spare the time, and the sedative before the effects of the motion begin to be felt. If once sea-sickness gains the ascendancy over the voyager, the time for remedies has gone by. There is nothing left for it but the old expedient of fearing that you will die for the first hour, and of fearing that you will not for the second. There is no other situation in life, unless it be the condemned cell without expectation of a reprieve, which so completely enables those who have passed through it to realise the meaning of hopelessness. The waves continue their ceaseless movement, which no one, in such conditions, is enthusiast enough to follow Æschylus in describing as "multitudinous laughter," the ship oscillates perpetually, and for the traveller there is no escape, until, in the words of the Psalmist, he "cometh to the haven where he would be." Once there, he will realise, with Paley, that there is no happiness equal to the cessation of misery, and will be fully prepared, with renewed health and rested brain, to endure the oscillation once more for the sake of once more realising the joy of an escape from it.

